# P-606. A Single-round Infectious Virus with Codon-deoptimized Genome: A Powerful Tool for Regulating Pathogenicity of WT/Live-Attenuated Viruses

**DOI:** 10.1093/ofid/ofae631.804

**Published:** 2025-01-29

**Authors:** Takafumi Noguchi, Anju Miyamori, Takeshi Sugimoto, Paola Miyazato, Ryo Sasaki, Sayuri Komatsu, Hirotaka Ebina

**Affiliations:** The research foundation for microbial diseases of Osaka university, Suita, Osaka, Japan; Osaka university, Suita, Osaka, Japan; The research foundation for microbial diseases of Osaka university, Suita, Osaka, Japan; The research foundation for microbial diseases of Osaka university, Suita, Osaka, Japan; The research foundation for microbial diseases of Osaka university, Suita, Osaka, Japan; The research foundation for microbial diseases of Osaka university, Suita, Osaka, Japan; Osaka university, Suita, Osaka, Japan

## Abstract

**Background:**

Coxsackievirus B3 (CVB3) is a ssRNA(+) virus of the *Picornaviridae* family associated with myocarditis and type I diabetes. Prevention of mother-to-child transmission is important, especially since the outcomes of neonatal infection can be fatal. However, vaccines have not yet been developed. In this study, we revealed that CVB3 codon-deoptimized in the nonstructural proteins’ region (CVB3_cd_) can be used as live-attenuated vaccines (LAVs). Furthermore, we showed that in co-infection experiments, a CVB3_cd_-based single-round infectious virus induces interference, significantly reducing the pathogenicity of viruses.

**Methods:**

CVB3_cd_ were designed to be enriched in CpG-containing rare codons in the 3CD region of the viral genome. Viral proliferation was evaluated based on plaque size and growth curve; and their pathogenicity in mice was determined by weight loss and survival after infection. Defective viral particles (DVPs) were generated by packaging WT or CVB3_cd_ replicons with a trans-expressed viral capsid protein. To evaluate the effect of viral interference with DVPs, WT or CVB3_cd_ were co-administered with the DVPs, and their pathogenicity in mice was monitored.

**Results:**

A decrease in viral replication was observed in correlation with the number of deoptimized codons, in addition to an increase in the survival rate of animals infected with the codon-deoptimized viruses. Among vaccination methods, intramuscular injection of the attenuated virus emerged as the least pathogenic option, effectively inducing neutralizing antibodies and providing protection against challenge by the WT virus. In addition, simultaneous infection of CVB3 with DVPs revealed that CVB3_cd_ replicon-based DVP (DVP_cd_), reduced the pathogenicity of CVB3 in mice and improved the safety of LAVs without compromising immunogenicity.

**Conclusion:**

Our data suggest that viral pathogenicity can be modulated by a combination of codon-deoptimization, route of administration, and viral interference. This approach could be a powerful strategy to develop effective and safe LAVs in a short period of time. Moreover, DVP_cd_ could be used as therapeutic tools against viruses due to their efficient virus-interfering effects.

**Disclosures:**

**Anju Miyamori, Master of Medicine**, The research foundation for microbial diseases of Osaka university: Grant/Research Support **Hirotaka Ebina, Doctor of Medicine**, The research foundation for microbial diseases of Osaka university: Grant/Research Support

